# Risk of recurrence in high-risk T1 colon cancer following endoscopic and surgical resection: registry-based cohort study

**DOI:** 10.1093/bjsopen/zrae053

**Published:** 2024-06-13

**Authors:** Emelie Nilsson, Erik Wetterholm, Ingvar Syk, Henrik Thorlacius, Carl-Fredrik Rönnow

**Affiliations:** Department of Clinical Sciences, Division of Surgery, Skåne University Hospital, Lund University, Malmö, Sweden; Department of Clinical Sciences, Division of Surgery, Skåne University Hospital, Lund University, Malmö, Sweden; Department of Clinical Sciences, Division of Surgery, Skåne University Hospital, Lund University, Malmö, Sweden; Department of Clinical Sciences, Division of Surgery, Skåne University Hospital, Lund University, Malmö, Sweden; Department of Clinical Sciences, Division of Surgery, Skåne University Hospital, Lund University, Malmö, Sweden

## Abstract

**Background:**

Endoscopic resection of T1 colon cancer (CC) is currently limited by guidelines related to risk of lymph node metastases. However, clinical outcome following endoscopic and surgical resection is poorly investigated.

**Method:**

A retrospective multicentre national cohort study was conducted on prospectively collected data from the Swedish colorectal cancer registry on all non-pedunculated T1 CC patients undergoing surgical and endoscopic resection between 2009 and 2021. Patients were categorized on the basis of deep submucosal invasion (Sm2–3), lymphovascular invasion (LVI), poor tumour differentiation, and R1/Rx into low- and high-risk cases. The primary outcomes of interest were recurrence rates and disease-free interval (DFI, defined as time from treatment to date of recurrence) according to resection methods and risk factors (sex, age at diagnosis, histologic grade, LVI, perineural invasion, mucinous subtype, submucosal invasion, tumour location, resection margin and nodal positivity in the surgical group).

**Results:**

In total, 1805 patients undergoing endoscopic (488) and surgical (1317) resection with 60.0 months median follow-up were included. Recurrence occurred in 18 (3.7%) endoscopically and 48 (3.6%) surgically resected patients. Adjuvant treatment was administered in 7.4% and 0.2% of the cases respectively in the surgical and endoscopically treated patients. Five-year DFI was 95.6% after endoscopic and 96.2% after surgical resection, with no significant difference when adjusting for confounding factors (HR 1.03, 95% c.i. 0.56 to 1.91, *P* = 0.920). There were no statistically significant differences in recurrence comparing endoscopic (1.7%) *versus* surgical (3.6%) low-risk and endoscopic (5.4%) *versus* surgical (3.8%) high-risk cases. LVI was the only significant risk factor for recurrence in multivariate Cox regression (HR 3.73, 95% c.i. 1.76 to 7.92, *P* < 0.001).

**Conclusions:**

This study shows no difference in recurrence after endoscopic and surgical resection in high-risk T1 CC. Although it was not possible to match groups according to treatment, the multivariate analysis showed that lymphovascular invasion was the only independent risk factor for recurrence.

## Teaser

Comparing endoscopically and surgically resected T1 colon cancer, we found no significant differences in recurrence rates, even in cases classified as high-risk according to current guidelines. The only significant risk factor for cancer recurrence was lymphovascular invasion.

## Introduction

Colon cancer (CC) constitutes approximately 70% of all colorectal cancer^1^ and advances in endoscopic resection techniques as well as implementation of screening programmes have increased the proportion of early CC amenable to endoscopic resection^2^. However, the majority of patients with T1 CC still undergo surgical resection, despite the benefits of bowel-preserving endoscopic resection^3^. Thus, the main factor hampering endoscopic resection as the final treatment is the risk of leaving concomitant lymph node metastases (LNM) untreated. Consequently, patients are categorized according to the risk of LNM as low or high risk, following endoscopic resection. European guidelines recommend subsequent surgery in high-risk cases, defined as presence of deep submucosal invasion (Sm2–3), lymphovascular invasion (LVI), high-grade cancer, tumour budding or R1/Rx resection^4^. Previous reports show that 6–16% of T1 CC harbour LNM^5–7^, but current guidelines categorize more than 60% of all T1 CC as high risk^8,9^, leading to surgical overtreatment when adhering to guidelines. This problem is also illustrated by the fact that only 5–15% of patients undergoing salvage surgery after endoscopic resection of high-risk T1 CC actually have LNM^10,11^. In this context, it is important to note that current treatment guidelines do not take clinical outcome into consideration and are solely based on studies investigating risk factors of LNM in surgically treated patients^12^. In fact, there is very limited knowledge in the literature on risk factors predicting recurrence in T1 CC, which are not necessarily the same used to predict LNM. For example, a recent study found no difference in recurrence after endoscopic submucosal dissection of high-risk T1 colorectal cancer, comparing subsequent surgery and surveillance only^13^. In addition, a previous study from our group showed that none of the risk factors used to predict LNM were risk factors of recurrence, and that rectal tumour location was the only factor predicting recurrence^14^. Based on the considerations above, the aim of this registry-based cohort study was to compare clinical outcomes after surgical and endoscopic resection of T1 CC and to investigate risk factors of recurrence. The research question was the difference in clinical outcome, in terms of recurrence and disease-free interval (DFI), comparing endoscopic and surgical resection of T1 CC.

## Material and methods

### Study design

This is a retrospective multicentre national cohort study on prospectively collected data conducted following STROBE and RECORD guidelines.

This study was approved by the Swedish Ethical Review Authority (2020-06676) prior to study start. The study was carried out in accordance with the Declaration of Helsinki. All data retrieved from the Swedish Colorectal Cancer Registry (SCRCR) were coded and anonymity was guaranteed.

### The Swedish Colorectal Cancer Registry and study population

All data were prespecified and retrieved from the SCRCR, a prospectively maintained national quality registry on colorectal cancer. The registry was introduced for CC in 2007 and contains data on preoperative staging, perioperative surgical details, postoperative histopathology, oncologic treatment and 5-year follow-up. Routine follow-up comprises clinical examination, blood test, CT scan of abdomen and thorax (1, 3 and 5 years) as well as colonoscopy (3 years). SCRCR has been shown to have high validity and coverage (99%) compared to the mandatory Swedish Cancer Registry^15^.

All patients with non-synchronous T1 CC undergoing surgical or endoscopic resection between January 2009 and March 2021 were identified in the SCRCR.

The following exclusion criteria were applied: neoadjuvant treatment, distant metastatic disease at index surgery, appendix tumours, patients awaiting/lost to follow-up, inconsistent information on tumour location and surgical approach as well as pedunculated tumours. Tumour morphology is not recorded in the SCRCR and cases reported as ‘not applicable’ under submucosal invasion were assumed to be pedunculated and excluded.

Patients were selected for endoscopic or surgical resection at the discretion of the managing surgeon and endoscopist based on lesion size, location, morphology and surface pattern, as well as level of endoscopic expertise and patient co-morbidities. These parameters are not recorded in the SCRCR. Biopsy-confirmed cancers were likely to be referred directly for surgery and patients undergoing subsequent surgical resection after endoscopic resection were selected in accordance with European Guidelines^4^ and are registered as surgically treated patients in the SCRCR.

### Outcomes of interest

The primary outcome measure was recurrence, comprising both local and distant recurrence. Local recurrence was defined as recurrence in the bowel segment or anastomosis as well as in mesocolic lymph nodes. DFI was defined as time from treatment to date of recurrence. Patients were censored at date of most recent follow-up, death or migration.

To compare clinical outcomes in the surgical and endoscopic groups, DFI and HR were analysed and adjusted for potential confounding factors. The time cut-off was set to 6 years to account for a delay in the routine 5-year follow-up appointment. Cumulative recurrence was also compared for surgically and endoscopically treated patients according to current risk classification. Low-risk cases were defined as absence of deep submucosal invasion (Sm2–3), LVI, poor tumour differentiation, as well as R1/Rx resection and high-risk groups were defined as harbouring at least one of these risk factors. This definition is consistent with current guidelines, except for not including tumour budding as a high-risk feature, because this parameter is not registered in the SCRCR^12,16^. Patients without risk factors but with missing data on any of the aforementioned variables could not reliably be classified as low risk and were labelled indeterminate. The high-, low- and indeterminate-risk groups were divided according to resection method and cumulative recurrence was compared between the groups.

Potential risk factors of recurrence analysed were sex, age at diagnosis, histologic grade, LVI, perineural invasion, mucinous subtype, submucosal invasion, tumour location and resection margin. Risk factor analyses were performed on the entire cohort (both surgical and endoscopic groups). N+ was analysed as a risk factor for recurrence in the surgical group separately. Histologic grade was classified as low- and high-grade cancer according to the Vienna classification and WHO guidelines^17,18^. LVI was defined as presence of lymphatic and/or vascular invasion. Submucosal invasion is reported in the SCRCR for non-pedunculated tumours according to Kudo, dividing the submucosal layer into upper (Sm1), middle (Sm2) and lower (Sm3) thirds. Resection margins were categorized as R0 and R1/Rx, comprising both lateral and vertical margins. Right colon was defined as caecum, ascending colon and transverse colon, and left colon was defined as descending and sigmoid colon.

### Statistical analyses

The surgical and endoscopic groups were compared in terms of DFI with Kaplan–Meier curves, log-rank test and Cox proportional hazard model, adjusted for potential confounding factors. The distribution of baseline characteristics in surgical and endoscopic patients was compared with Fisher’s exact test/Freeman–Halton extension, chi-square test and Mann–Whitney *U*-test when appropriate. Risk factor analyses were performed with uni- and multivariate Cox proportional hazard models, with HR as the effect measure and 95% c.i. Kaplan–Meier curves were plotted for each factor and the assumptions of proportionality were considered met in all factors except for perineural invasion and resection margin; hence, these were not included in the Cox regression model. Missing values were imputed using the Mersenne twister random number generator, and 20 imputations were performed. Sensitivity analyses, with list-wise deletion, were performed. Incidence of recurrence was compared in N0 and N+ patients in the surgical group with a chi-square test. Time from resection to recurrence was compared with the Mann–Whitney *U*-test in endoscopically and surgically treated patients. *P* < 0.05 was considered statistically significant. Analyses were performed in IBM SPSS version 28.0 (SPSS Inc., Chicago, IL, USA).

## Results

### Study population

**Fig. 1:**
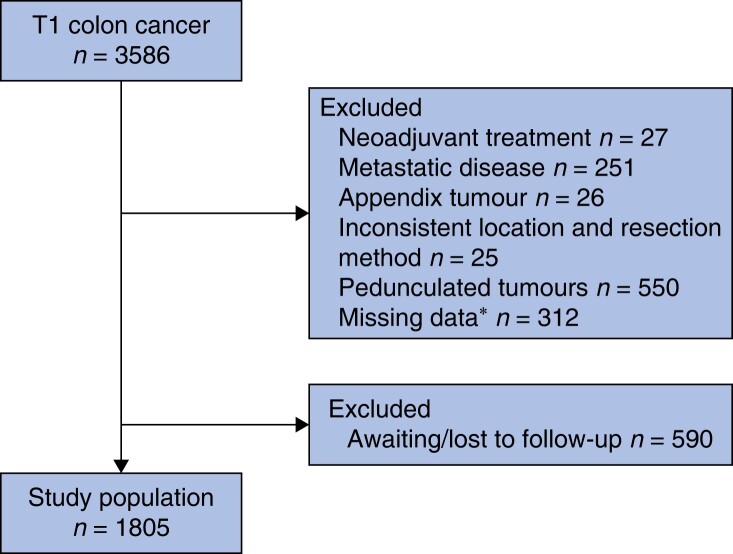
Flowchart of inclusion and exclusion *Missing data on neoadjuvant treatment and metastatic disease.

A total of 3586 patients with endoscopically and surgically treated T1 CC were identified. After applying our exclusion criteria, 1805 patients remained for analyses and comprised the study population (*Fig. 1*). In all, 1317 (73.0%) patients underwent surgical resection and 488 (27.0%) patients underwent endoscopic resection. Median age at diagnosis was 72 years in the surgical group and 73 years in the endoscopic group and 48.7% of all patients were female, with similar gender distribution in both groups. Incidence of LNM was 154/1317 (11.7%) and median number of harvested lymph nodes was 16 (i.q.r. 12–23) in the surgical group. Tumours were more frequently located in the left colon in the endoscopic (89.8%) compared to the surgical group (51.2%) and high-risk lesions were more frequent in the surgical group (63.5%) compared to the endoscopic group (30.3%). Adjuvant chemotherapy was given to 97 (7.4%) of the 1317 surgically treated patients and one (0.2%) of the 488 endoscopically treated patients. Median follow-up was 60.0 months (i.q.r. 38.0–63.4), and 66 (3.7%) recurrences were detected in the entire cohort, of which 9 were local, 51 distant, five both local and distant and one unspecified. Local recurrence was more frequent in the endoscopic (16.7%) compared to the surgical (12.5%) group. Baseline characteristics of the study population are summarized in *Table 1*.

**Table 1:** Baseline characteristics of T1 colon cancer study population

	Surgical resection	Endoscopic resection	*P*
	(total = 1317)	(total = 488)	
**Sex**			
Male	671 (50.9)	255 (52.3)	0.622
Female	646 (49.1)	233 (47.7)	
Age at diagnosis (years), median (i.q.r.)	72 (65–79)	73 (64.5–79)	0.454
**N stage**			
N0	1144 (86.9)	–	–
N+	154 (11.7)	–	
Nx	18 (1.4)	–	
Missing	1 (0.1)	–	
**Tumour location**			
Right colon	641 (48.7)	50 (10.2)	<0.001
Left colon	674 (51.2)	438 (89.8)	
Missing	2 (0.2)	0	
**Recurrence location**			
Total *n* recurrence	48	18	
Local*	6 (12.5)	3 (16.7)	<0.001
Distant	41 (85.4)	10 (55.6)	
Both	0	5 (27.8)	
Missing	1 (2.1)	0	
**Risk group** †			
Low risk	329 (25.0)	118 (24.2)	<0.001
High risk	836 (63.5)	148 (30.3)	
Indeterminate	152 (11.5)	222 (45.5)	
**Adjuvant chemotherapy**			
No	1220 (92.6)	487 (99.8)	<0.001
Yes	97 (7.4)	1 (0.2)	

Values are *n* (%) unless otherwise stated.

*Local recurrence comprise recurrence in bowel segment and mesenteric lymph nodes. †Low risk: absence of deep submucosal invasion (Sm2–3), lymphovascular invasion, poor tumour differentiation (high-grade cancer) and R1/RX resection. High risk: one or more of the previous high-risk features present. Indeterminate: missing values prohibiting low-risk classification.

### Clinical outcome

**Fig. 2:**
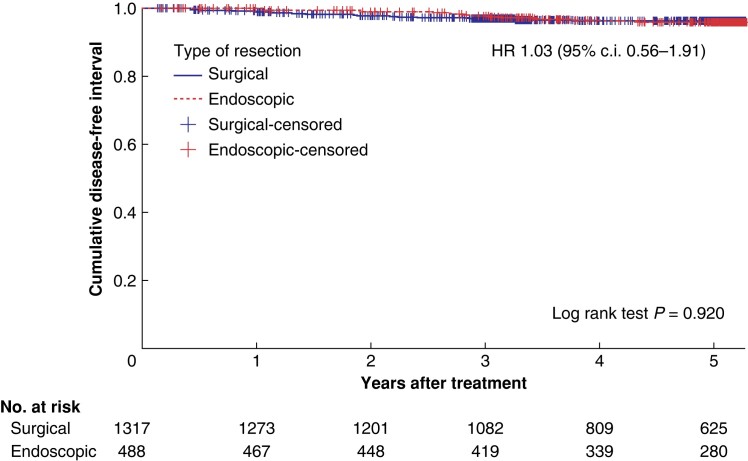
Kaplan–Meier curve showing recurrence after surgical and endoscopic resection

Three- and five-year DFI were 97.1% and 96.2% in the surgical group and 97.8% and 95.6%, in the endoscopic group respectively. There was no significant difference in DFI between the surgical and endoscopic groups tested with Cox regression analysis, adjusted for confounding factors (HR 1.03, 95% c.i. 0.56 to 1.91, *P* = 0.920; *Fig. 2*). Incidence of recurrence during the study period was 3.6% in the surgical and 3.7% in the endoscopic group (*Table 1*). Recurrences were local in six of 48 (12.5%) and three of 18 (16.7%) in the surgical and endoscopic groups respectively, and additionally, five of 18 (27.8%) patients were diagnosed with combined local and distant recurrence in the endoscopic group (*Table 1*). Median time to recurrence was 18.6 months (i.q.r. 11.7–34.1) in the surgical group and 34.1 (i.q.r. 23.2–41.2) months in the endoscopic group (*P* = 0.007).

**Fig. 3:**
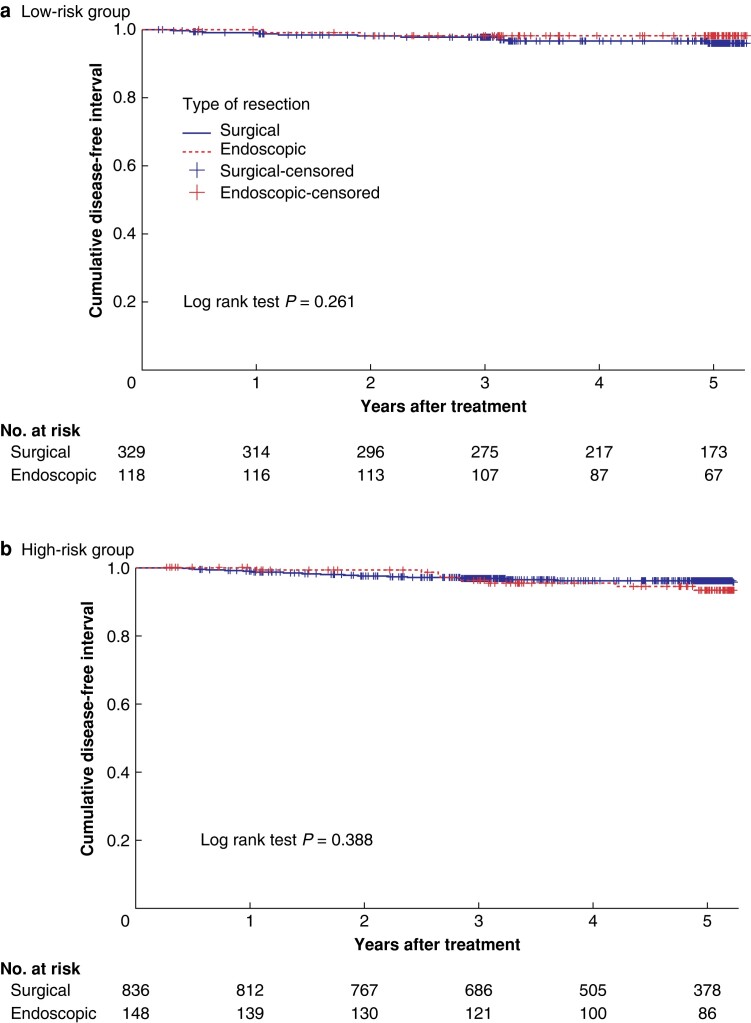
**a**. Kaplan–Meier curve showing recurrence after surgical and endoscopic resection in low-risk group. **b.** Kaplan–Meier curve showing recurrence after surgical and endoscopic resection in high-risk group

Patients were divided into high-, low- and indeterminate-risk groups. In the endoscopic group, 30.3% were high risk, 24.2% were low risk and 45.5% were indeterminate. In the surgical group, 63.5% were high risk, 25.0% were low risk and 11.5% were indeterminate. Cumulative incidence of recurrence did not differ significantly, comparing surgically and endoscopically treated cases, stratified according to risk group (*Table 2*). Kaplan–Meier curves did not show any difference in DFI, comparing surgically and endoscopically treated low- and high-risk patients (*Fig. 3a*, *and b*).

**Table 2:** Recurrence according to resection method and risk group in T1 colon cancer

	*n* of total
**Low risk**	
Surgical resection	12 of 329 (3.6)
Endoscopic resection	2 of 118 (1.7)
**High risk**	
Surgical resection	32 of 836 (3.8)
Endoscopic resection	8 of 148 (5.4)
**Indeterminate**	
Surgical resection	4 of 152 (2.6)
Endoscopic resection	8 of 222 (3.6)

Values are *n* of total (%).

Low risk: absence of deep submucosal invasion (Sm2–3), LVI, poor tumour differentiation (high-grade cancer) and R1/RX resection. High risk: one or more of the previous high-risk features present. Indeterminate: missing values prohibiting low-risk classification.

### Risk factors for recurrence

Incidence of recurrence according to depth of submucosal invasion was 3.7% in Sm1, 3.8% in Sm2 and 3.2% in Sm3 tumours (*Table 3*). High- and low-grade cancer had 7.0% and 3.7% recurrence respectively. Recurrence was 6.3% when mucinous subtype was present and 3.5% when absent. Neither deep submucosal invasion, high-grade cancer nor mucinous subtype were significant risk factors for recurrence in uni- and multivariate Cox regression analysis (*Table 4*). In fact, LVI was the only statistically significant risk factor for recurrence in both uni- and multivariate Cox regression analyses (*Table 4*). Incidence of recurrence according to LVI was 10.0% when present and 2.9% when absent (*Table 3*). Incidence of recurrence was significantly higher in N+ patients (10.4%) compared with N0 patients (2.7%) (*P* < 0.001) in the surgical group. R0 resection resulted in 3.6% and R1/Rx resection resulted in 5.3% recurrence, and perineural invasion resulted in 16.7% recurrence when present and 3.3% when absent. Resection margin and perineural invasion were not included as parameters in the Cox regression analysis because the assumptions of proportionality were not considered met.

**Table 3:** Recurrence according to potential risk factors in T1 colon cancer

	Number of patients *n*	Recurrence *n* (%)
**Resection**		
Surgical	1317	48 (3.6)
Endoscopic	488	18 (3.7)
**Sex**		
Male	926	30 (3.2)
Female	879	36 (4.1)
**Age at diagnosis (years)**		
<50	76	4 (5.3)
50–59	161	2 (1.2)
60–69	499	19 (3.8)
70–79	666	23 (3.5)
≥80	403	18 (4.5)
**Histologic grade**		
Low grade	1452	53 (3.7)
High grade	115	8 (7.0)
Missing	238	5 (2.1)
**Lymphovascular invasion**		
No	1363	39 (2.9)
Yes	110	11 (10.0)
Missing	332	16 (4.8)
**Perineural invasion**		
No	1400	46 (3.3)
Yes	18	3 (16.7)
Missing	387	17 (4.4)
**Mucinous tumour**		
No	1467	52 (3.5)
Yes	111	7 (6.3)
Missing	227	7 (3.1)
**Submucosal invasion**		
Sm1	730	27 (3.7)
Sm2	368	14 (3.8)
Sm3	495	16 (3.2)
Missing	212	9 (4.2)
**N stage**		
N0	1144	31 (2.7)
N+	154	16 (10.4)
Nx	18	1 (5.6)
Missing	1	0
**Location**		
Right colon	691	25 (3.6)
Left colon	1112	41 (3.7)
Missing	2	0
**Resection margin**		
R0	1649	60 (3.6)
R1/Rx	76	4 (5.3)
Missing	80	2 (2.5)

**Table 4:** Uni- and multivariate Cox regression analyses on potential risk factors of recurrence in T1 colon cancer

	Incidence*	Univariate analyses			Multivariate analyses		
	/10 000	HR	95% c.i.	*P*	HR	95% c.i.	*P*
**Resection**							
Surgical	86	1.00	Ref.		1.00	Ref.	
Endoscopic	83	0.97	0.57,1.68	0.924	1.03	0.56,1.91	0.920
**Sex**							
Male	75	1.00	Ref.		1.00	Ref.	
Female	95	1.25	0.77,2.04	0.359	1.26	0.77,2.07	0.352
Age at diagnosis	–	1.01	0.99,1.03	0.473	1.00	0.98,1.03	0.762
**Histologic grade**							
Low grade	84	1.00	Ref.		1.00	Ref.	
High grade	172	2.09	0.99,4.38	0.052	1.37	0.59,3.15	0.464
**Lymphovascular invasion**							
No	66	1.00	Ref.		1.00	Ref.	
Yes	251	3.96	1.97,7.93	<0.001	3.73	1.76,7.92	<0.001
**Mucinous tumour**							
No	82	1.00	Ref.		1.00	Ref.	
Yes	158	1.97	0.87,4.46	0.106	1.75	0.74,4.15	0.201
**Submucosal invasion**							
Sm1	85	1.00	Ref.		1.00	Ref.	
Sm2	88	1.09	0.57,2.07	0.799	1.06	0.56,2.04	0.853
Sm3	75	0.95	0.51,1.76	0.864	0.89	0.46,1.71	0.721
**Tumour location**							
Right colon	87	1.00	Ref.				
Left colon	83	0.96	0.59,1.59	0.884	1.07	0.62,1.85	0.802

*Number of recurrences per 10 000 person-year at risk.

### Sensitivity analyses and missing data

Multiple imputations were performed to account for missing data on risk factors. Overall, 5.6% of all values were missing. In risk factor analyses and DFI comparisons using Cox proportional hazard regression, sensitivity analyses with list-wise deletion were performed. There was no difference in significance and HRs were similar when comparing data with complete cases and imputed data (*Supplementary Materials*).

## Discussion

Endoscopic resection of T1 CC is a desirable treatment option offering reduced morbidity and mortality rates and cost compared with surgery^19,20^. These benefits are currently balanced against the risk of concomitant LNM determining resection method according to prevailing guidelines^12^. However, clinical outcome following endoscopic and surgical resection of T1 CC is elusive and not acknowledged in current guidelines. This multicentre retrospective cohort study shows that endoscopic and surgical resection result in equal incidence of recurrence in not only low- but also high-risk patients. Thus, circumventing current guidelines and evading subsequent surgery in endoscopically treated high-risk T1 CC did not have a negative effect on incidence of recurrence. It is noteworthy that LVI was identified as the only significant and independent risk factor for cancer recurrence, irrespective of resection method. The overall incidence of recurrence in T1 CC was 3.7% during the 60.0 months (median) follow-up period. When compared, incidence of recurrence and 5-year DFI were almost identical in the surgical and endoscopic groups. Indeed, the two groups differed in terms of baseline characteristics and risk factors were overall more frequent in surgical cases. However, when adjusting for potential confounding factors, there was still no statistically significant difference in risk of recurrence. Lesions were thereafter divided according to current guidelines into high- and low-risk groups. Intriguingly, 148 (30.3%) of the endoscopic resected patients were high risk and should have undergone subsequent surgical resection, according to guidelines. However, there was no statistically significant difference comparing the endoscopic with the corresponding surgical high-risk group, with 5.4% and 3.8% recurrence respectively. The literature on comparisons of endoscopic and surgical resection of T1 tumours is heterogeneous and in part conflicting. Nevertheless, some previous studies have shown similar recurrence rates after endoscopic and surgical resection of T1 colorectal cancer, lending support to our findings^13,21^. In contrast, the majority of studies investigating high-risk T1 colorectal cancer have reported a higher incidence of recurrence after endoscopic resection, ranging from 7% to 15%, compared with surgical resection, ranging from 2% to 4%^22–26^. However, these studies are in general of limited size, including a maximum of 106 endoscopic high-risk resections^22^, and often combine the results of T1 colon and rectal cancer^21,24^. This is problematic because a growing body of evidence suggests that both incidence of LNM^5,27,28^ and recurrence^14,29^ are lower in T1 colon compared with rectal cancer. Nevertheless, another author reported CC separately and found that cancer recurrence was similar in endoscopically and surgically treated high-risk patients, which is in line with our findings^22^. Moreover, in the present study, there was no significant difference in recurrence after endoscopic (1.7%) and surgical (3.6%) resection of low-risk tumours. This finding is not controversial and aligned with current treatment guidelines and previous studies reporting low and comparable risks of recurrence after endoscopic and surgical resection in the absence of risk factors^24,25,30^. Of note, a third, indeterminate group was included in our analysis, where classification into high or low risk was not possible due to missing values. Incidence of recurrence in the indeterminate groups did not differ significantly and was 3.6% and 2.6% after endoscopic and surgical resection respectively. It is important to note that 45.5% of all endoscopic resections were indeterminate, presumably reflecting the quality of the endoscopic specimens, disenabling correct pathologic assessment. However, the recurrence rate in the endoscopic indeterminate group in relation to the low- and high-risk groups indicates that this group in fact is a mix of both low- and high-risk lesions. Taken together, there was no difference in recurrence comparing surgically and endoscopically treated T1 CC regardless of risk factors and current risk classification. Interestingly, median time from resection to recurrence was 18.6 and 34.1 months in the surgical end endoscopic groups respectively. This finding is hard to explain and contrasts several publications showing no difference in time from resection to recurrence, following endoscopic and surgical resection^21,29^. Nevertheless, our finding is supported by a previous study, reporting that time to recurrence was 37.5 months after endoscopic and 20.0 months after surgical resection^30^. In this context, it is important to note that previous studies are in general of limited size and recurrences are consequently rare events, making analyses of median time to recurrence uncertain. Reliably identifying patients with an increased risk of negative clinical outcomes is important to ensure optimal management of patients with early CC. In the present study, we found that LVI was the only independent risk factor for recurrence. Thus, LVI was a predictor of recurrence regardless of resection method, and incidence of recurrence increased from 2.9% when LVI was absent to 10.0% when LVI was present. Numerous studies have consistently identified LVI as a strong and independent predictor of both LNM^9,31,32^ and recurrence^24,33^, in support of our findings. In this context it is interesting to note that recurrences occurred in 10.2% of N+ and 2.8% of N0 surgically treated patients, emphasizing that LNM is a prognostic marker of poor outcome. Furthermore, depth of submucosal invasion has been a cornerstone in the decision process of endoscopically treated T1 CC for decades. Herein, depth of submucosal invasion was not a significant risk factor for recurrence and incidence of recurrence was almost identical in Sm1, 2 and 3 tumours. This finding is not controversial and is supported by several studies showing that deep submucosal invasion is not a risk factor for recurrence in T1 colorectal cancer^14,24,29,31^. In addition, recent studies have also questioned submucosal invasion as an independent risk factor for LNM^32,34^. Hence, it is evident that maintaining submucosal invasion as a risk factor in treatment recommendations results in a substantial risk of surgical overtreatment, especially when considering that most T1 CC are Sm2–3, as shown herein. Moreover, high-grade cancer—that is, poor tumour differentiation—was not a significant risk factor for recurrence in our study. In addition, incidence of recurrence only differed slightly comparing R0 resected patients (3.6% recurrence) with R1/RX cases (5.3% recurrence), although resection margin was not included as a risk factor in our Cox regression analysis because proportionality was not met. In contrast, several studies have shown increased risk of recurrence in high-risk tumours, which includes both R1/RX resection margin and high-grade cancer^22,24^. However, the actual importance of these two risk factors is elusive because they are consequently combined with deep submucosal invasion, LVI and sometimes tumour budding in the literature. Additionally, previous studies analysed colorectal cancer combined and the proportion of rectal lesions will also presumably affect the incidence of recurrence, as stated previously. This study contains certain limitations. First, the design was retrospective and not randomized, with the risk of selection bias. Additionally, we were unable to adjust for tumour size and co-morbidities, which constitute the most important factors that could address the risk of selection bias. Thus, it is possible that lesions in the endoscopic group were overall smaller than in the surgical group and it is also possible that patients in the endoscopic group had a higher degree of disease burden, especially patients with high-risk features. Second, data on method of endoscopic resection and en-bloc/piecemeal resection, which could influence the rate of recurrence, could not be retrieved. In addition, our data contain missing values, constituting another limitation. However, imputation was performed to account for missing data in the Cox regression model, and patients with missing data, prohibiting classification into high-/low-risk groups, were analysed as indeterminate. Additionally, sensitivity analyses showed no difference in significance and HR levels, comparing complete and imputed data. Finally, tumour budding is not registered in the SCRCR and was neither included in our multivariate regression analyses nor as a parameter in our risk group stratification. Consequently, cases in the low-risk surgical and endoscopic groups could potentially have been high-risk tumours according to prevailing guidelines. This could explain the fact that recurrence was more common in the low-risk surgical *versus* endoscopic group.

In conclusion, there was no difference in recurrence after endoscopic resection of not only low- but also high-risk T1 CC compared to surgical resection. Although it was not possible to match groups according to treatment, the multivariate analysis showed that LVI was the only risk factor predicting cancer recurrence.

## Supplementary Material

zrae053_Supplementary_Data

## Data Availability

Data are available from the Swedish Colorectal Cancer Registry.
