# Supplementary material for: Risk of recurrence in high-risk T1 colon cancer following endoscopic and surgical resection: registry-based cohort study
Source: BJS Open. 2024 Jun 13;8(3):zrae053. doi: 10.1093/bjsopen/zrae053 (PMC11170496; doi:10.1093/bjsopen/zrae053)
Supplement: zrae053_Supplementary_Data [file zrae053_supplementary_data.docx]

Risk of recurrence in high-risk T1 colon cancer following endoscopic and surgical resection*: A registry-based cohort study*

Authors

Emelie Nilsson (MD), Erik Wetterholm (MD), Ingvar Syk (MD, PhD), Henrik Thorlacius (MD, PhD) and Carl-Fredrik Rönnow (MD, PhD)*

Affiliation

Department of Clinical Sciences, Malmö, Division of Surgery, Skåne University Hospital, Lund University, Malmö, Sweden.

**Corresponding author.** Carl-Fredrik Rönnow **ORCID ID**; **0000-0001-5848-1407**

**Supplementary Materials - Index**

| Sensitivity analysis - Cox proportional hazard regression, list-wise deletion | *Page 2* |
| --- | --- |

| **Supplementary Table**   \|  \|  \|  \|  \|  \| Multivariate analyses \|  \| \| --- \| --- \| --- \| --- \| --- \| --- \| --- \| \|  \|  \|  \|  \| HR \| 95% CI \| *p*-value \| \|  \|  \|  \|  \|  \|  \|  \| \| Resection \| Surgical \|  \|  \| 1.00 \| Ref. \|  \| \|  \| Endoscopic \|  \|  \| 0.86 \| 0.31-2.36 \| 0.763 \| \|  \|  \|  \|  \|  \|  \|  \| \| Gender \| Male \|  \|  \| 1.00 \| Ref. \|  \| \|  \| Female \|  \|  \| 1.11 \| 0.60-2.06 \| 0.729 \| \|  \|  \|  \|  \|  \|  \|  \| \| Age at diagnosis \|  \|  \|  \| 1.00 \| 0.97-1.02 \| 0.777 \| \|  \|  \|  \|  \|  \|  \|  \| \| Histologic grade \| Low-grade \|  \|  \| 1.00 \| Ref. \|  \| \|  \| High-grade \|  \|  \| 1.64 \| 0.65-4.16 \| 0.297 \| \|  \|  \|  \|  \|  \|  \|  \| \| Lymphovascular invasion \| No \|  \|  \| 1.00 \| Ref. \|  \| \|  \| Yes \|  \|  \| 2.83 \| 1.26-6.31 \| 0.011 \| \|  \|  \|  \|  \|  \|  \|  \| \| Mucinous tumour \| No \|  \|  \| 1.00 \| Ref. \|  \| \|  \| Yes \|  \|  \| 1.26 \| 0.43-3.71 \| 0.681 \| \|  \|  \|  \|  \|  \|  \|  \| \| Submucosal invasion \| Sm1 \|  \|  \| 1.00 \| Ref. \|  \| \|  \| Sm2 \|  \|  \| 1.21 \| 0.58-2.51 \| 0.616 \| \|  \| Sm3 \|  \|  \| 0.65 \| 0.31-1.40 \| 0.276 \| \|  \|  \|  \|  \|  \|  \|  \| \| Tumour location \| Right colon \|  \|  \|  \|  \|  \| \|  \| Left colon \|  \|  \| 0.94 \| 0.49-1.81 \| 0.86 \| \|  \|  \|  \|  \|  \|  \|  \|   Supplementary table shows multivariate list-wise deletion analysis of risk factors of recurrence. |  |
| --- | --- | --- | --- | --- | --- | --- | --- | --- | --- | --- | --- | --- | --- | --- | --- | --- | --- | --- | --- | --- | --- | --- | --- | --- | --- | --- | --- | --- | --- | --- | --- | --- | --- | --- | --- | --- | --- | --- | --- | --- | --- | --- | --- | --- | --- | --- | --- | --- | --- | --- | --- | --- | --- | --- | --- | --- | --- | --- | --- | --- | --- | --- | --- | --- | --- | --- | --- | --- | --- | --- | --- | --- | --- | --- | --- | --- | --- | --- | --- | --- | --- | --- | --- | --- | --- | --- | --- | --- | --- | --- | --- | --- | --- | --- | --- | --- | --- | --- | --- | --- | --- | --- | --- | --- | --- | --- | --- | --- | --- | --- | --- | --- | --- | --- | --- | --- | --- | --- | --- | --- | --- | --- | --- | --- | --- | --- | --- | --- | --- | --- | --- | --- | --- | --- | --- | --- | --- | --- | --- | --- | --- | --- | --- | --- | --- | --- | --- | --- | --- | --- | --- | --- | --- | --- | --- | --- | --- | --- | --- | --- | --- | --- | --- | --- | --- | --- | --- | --- | --- | --- | --- | --- | --- | --- | --- | --- | --- | --- | --- | --- | --- | --- | --- | --- | --- | --- | --- | --- | --- | --- |
|  |  |
|  |  |
